# Regulation of Treg Cell Metabolism and Function in Non-Lymphoid Tissues

**DOI:** 10.3389/fimmu.2022.909705

**Published:** 2022-06-02

**Authors:** Kai Yang

**Affiliations:** ^1^ Department of Pediatrics and the Herman B Wells Center for Pediatric Research, Indiana University School of Medicine, Indianapolis, IN, United States; ^2^ Department of Microbiology and Immunology, Indiana University School of Medicine, Indianapolis, IN, United States

**Keywords:** tissue Treg cells, Treg metabolism, Treg homeostasis, Treg function, metabolic signaling

## Abstract

Regulator T cells (Tregs) play pivotal roles in maintaining immune tolerance and regulating immune responses against pathogens and tumors. Reprogramming of cellular metabolism has been determined as a crucial process that connects microenvironmental cues and signaling networks to influence homeostasis and function of tissue Tregs. In adaptation to a variety of non-lymphoid tissues, Tregs coordinate local immune signals and signaling networks to rewire cellular metabolic programs to sustain their suppressive function. Altered Treg metabolism in turn shapes Treg activation and function. In light of the advanced understanding of immunometabolism, manipulation of systemic metabolites has been emerging as an attractive strategy aiming to modulate metabolism and function of tissue Tregs and improve the treatment of immune-related diseases. In this review, we summarize key immune signals and metabolic programs involved in the regulation of tissue Tregs, review the mechanisms underlying the differentiation and function of Tregs in various non-lymphoid tissues, and discuss therapeutic intervention of metabolic modulators of tissue Tregs for the treatment of autoimmune diseases and cancer.

## Introduction

Tregs expressing forkhead box 3 (FOXP3), a specialized subset of CD4^+^ T cells, play crucial roles in maintaining immune tolerance and preventing autoimmunity (1). Tregs originally develop in the thymus, termed thymus-derived Tregs (tTregs), but may also generate at peripheral tissues known as peripherally-derived Tregs (pTregs) (2). Aside from the divergence of their origin and location (lymphoid and non-lymphoid tissues), tTregs and pTregs display heterogeneous T cell receptor (TCR) repertoires recognizing diverse self- and non-self-antigens to preserve immune homeostasis of lymphoid and non-lymphoid tissues. In terms of activation and differentiation, Tregs can be divided into two distinct subsets: central Tregs (cTregs) and effector Tregs (eTregs) (3, 4). cTregs express the lymphoid homing molecules CD62L and CC-chemokine receptor 7 (CCR7) and apparently locate in the lymphoid tissues (3). In contrast, eTregs upregulate expression of activation-associated markers such as CD44 and inducible costimulatory (ICOS) (3), which primarily reside in non-lymphoid tissues. A growing body of evidence shows that diverse immune signals (like TCR, co-stimulatory, and cytokine signals) in the tissue microenvironment orchestrate the differentiation and maintenance of eTregs through activating various signaling pathways such as mechanistic target of rapamycin (mTOR) signaling pathway and diverse transcription factors (5, 6). Cooperation of FOXP3 with various transcription factors confers Tregs orchestrate distinct suppressive programs targeting diverse immune responses in a variety of non-lymphoid tissues (7). Single-cell transcriptomic and chromatin accessibility analyses indicate that Tregs establish unique transcriptional features in adaptation to non-lymphoid tissues in health and disease (8–12).

Reprogramming of cellular metabolism in Tregs represents a key process that underlies Treg functional stability and specification in adaptation to non-lymphoid tissues (13, 14). It is now clear that Tregs and effector helper CD4^+^ T cells utilize distinct metabolic programs (15). In contrast to effector helper CD4^+^ T cells that mainly use aerobic glycolysis for their expansion and function, Tregs largely rely on mitochondrial oxidative phosphorylation (OXPHOS) and fatty acid oxidation (FAO) to fulfill their bioenergetic demands and suppressive function. Dysregulation of mitochondrial metabolism and glycolysis is detrimental for Tregs in preserving immune homeostasis in a variety of non-lymphoid tissues (16). In addition to intracellular metabolism, extracellular metabolites produced in distinct tissues provide unique signals to orchestrate functional diversification of Tregs in different non-lymphoid tissues. It is well-established that Tregs in the visceral adipose tissue (VAT-Tregs) display active proliferation with profound effects on local and systemic metabolism (17). In this lipid-rich organ, VAT-Tregs acquire unique transcriptional programs characterized by increased capacity for lipid biosynthesis and uptake of long-chain fatty acids (LCFAs) (17). In contrast, colonic Tregs reside in the microenvironment with high levels of short-chain fatty acids (SCFAs), which promote their differentiation and suppressive function to prevent the development of colitis (18–20). It is conceivable that diverse immune signals and metabolites available in non-lymphoid tissues constitute distinct physiological niches that shape the differentiation and accumulation of eTregs *via* rewiring cellular signaling and metabolic networks (6). In this review, we will first describe and summarize key immune signals and signaling pathways involved in regulating Treg cell metabolism and function; Second, we will provide an overview on intracellular metabolic programs underlying homeostasis and functional diversification of Tregs in different non-lymphoid tissues; lastly, we will discuss therapeutic manipulation of systemic metabolites to modulate tissue Treg function for the treatment of autoimmune disease and cancer.

## Key Immune Signals and Signaling Pathways Underlying Treg Metabolism and Function

**Figure 1:**
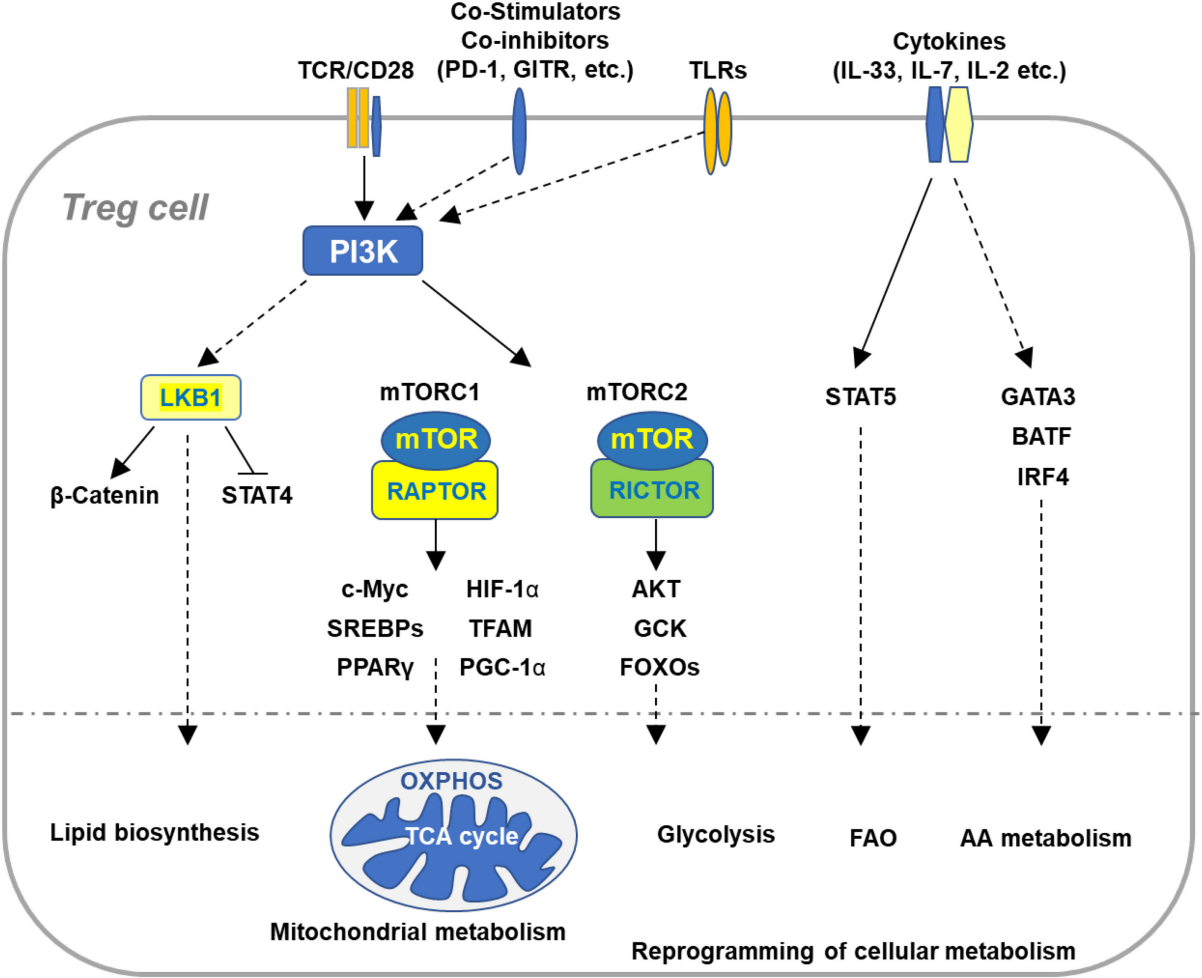
Immune signaling pathways involved in the regulation of Treg cell metabolism and adaptation to non-lymphoid tissues. Microenvironmental antigens, co-stimulators, TLRs, and cytokines orchestrate key immune signaling pathways to reprogram cellular metabolism of Tregs in adaptation to non-lymphoid tissues. The PI3K signaling pathway senses the immune signals derived from TCR, co-stimulatory receptors, co-inhibitory receptors, and TLRs, which in turn activates mTOR complexes to orchestrate reprogramming of cellular metabolism *via* various transcription factors. TCR and co-stimulation activates LKB1 signaling pathway that restricts STAT4 function and promotes stabilization of β-catenin and metabolic reprogramming of Tregs in maintaining immune homeostasis of multiple non-lymphoid tissues. In response to TCR and co-stimulatory signals in the tissue microenvironment, Tregs coordinate a variety of transcription factors that upregulate expression of the genes involved in diverse metabolic programs, including lipid biosynthesis, mitochondrial metabolism, glycolysis, FAO, and AA metabolism. Cytokines existed in various tissues sustain homeostasis and accumulation of tissue Tregs through different transcription factors. VAT-Treg cell accumulation is dependent upon TCR, FOXP3, and IL-33 signaling through activation of GATA3, IRF4, and BATF. IL-7 is required to maintain homeostasis and function of skin-Tregs. TCR, T cell receptor; FAO, fatty acid oxidation; AA, amino acids; VAT, visceral adipose tissue.

The development of Tregs expressing the lineage transcription factor FOXP3 is driven by high-affinity TCR signals in the thymus (21–23). Continuous TCR signaling is required to sustain Foxp3 expression and suppressive function of mature Tregs in the periphery (24, 25), in line with the notion that continued expression of FOXP3 enforces functional integrity of Tregs (26). Later studies indicate that TCR signaling coordinates reprogramming of intracellular metabolism to dictate the formation of cTregs and eTregs (27, 28), representing two Treg subsets with distinct patterns of cell migration and tissue localization (3). It is important to note that non-lymphoid tissues contain high proportions of eTregs (3), implying that microenvironmental immune signals, such as diverse antigens and cytokines derived from the tissues, promote eTreg cell formation and survival. Here, we will summarize and discuss key immune signals and signaling pathways that orchestrate homeostasis and function of tissue Tregs (**Figure 1**).

### TCR and Co-Stimulatory/Co-Inhibitory Signals

Continuous TCR signaling is required to sustain the generation and function of eTregs (24). Self-antigens in non-lymphoid tissues activate TCR signaling in eTregs (3). It is well-established that TCR stimulation activates phosphoinositide 3-kinase (PI3K)/protein kinase B (AKT) signaling pathway in Tregs. PI3K catalyzes the conversion of PtdIns-4,5-P_2_ (PIP2) toward PtdIns-3,4,5-P_3_ (PIP3), which in turn activates AKT (29). Knock-in of kinase-inactive p110δ reduces proportions of Tregs leading to the development of colitis (30, 31). Inactivation of p110δ also impairs Treg-mediated repression of antitumor immunity (32). As a key kinase downstream of PI3K, AKT is tightly controlled. Tregs display less phosphorylation of AKT at serine (S) 473 with a concomitant reduction of phosphorylated forkhead box transcription factors (FOXOs) (33), known as the substrates of AKT. In line with hypoactivation of AKT in mature Tregs, FOXOs are crucial to establish and sustain suppressive function of Tregs (34–36). Conversely, Treg-specific ablation of phosphatase and tensin homolog (PTEN) constitutively activates AKT at S473, which enhances phosphorylation of FOXOs and in turn compromises their transcriptional function (37). Consequently, PTEN-deficient Tregs show impairment of functional stability and fail to prevent autoimmunity (38). A further study indicated that constitutive activation of FOXO1 in Tregs disrupt their metabolic reprogramming and mTOR activation, causing the development of severe autoinflammation in multiple tissues (39). These studies suggest that precise regulation of the PI3K/AKT/FOXOs axis is crucial for tissue Tregs in maintaining their homeostasis and function in response to local TCR signals.

Aside from local TCR signals, tissue Tregs receive signals derived from a variety of co-stimulatory/co-inhibitory receptors to maintain proper function and metabolism. CD28 is the well-known costimulatory molecules required for T cell activation. It has been reported that CD28 plays an intrinsic role in maintaining Treg cell homeostasis and function (40). Treg-specific ablation of CD28 enhances homeostatic proliferation of Tregs, suggestive of their altered reprogramming of cellular metabolism. Loss of CD28 impairs Treg cell function, leading to spontaneous development of inflammatory responses in the skin and lung (40). A following study revealed that CD28 is important to drive the differentiation of cTregs into eTregs (41). Despite that loss of CD28 has no substantial impact on numbers of Tregs, it impairs homing of Tregs to a variety of non-lymphoid tissues. Tregs constitutively express the costimulatory receptor ICOS that provides signaling to suppress accumulation and suppressive function of Tregs in the visceral adipose tissue (42). In contrast, co-inhibitory receptors mediate signals to restrain Treg metabolism and function. An emerging study showed that the co-inhibitory receptor programmed death-1 (PD-1) suppresses Treg cell activation and function through altering metabolic fitness (43). Specific depletion of PD-1 in Tregs potentiates their suppressive function and ameliorates the progression of experimental autoimmune encephalomyelitis (EAE) (43). In line with the observation, blockade of PD-1 signaling enhances proliferation and suppressive function of intratumoral Tregs, facilitating cancer immunoevasion (44). In addition, Tregs constitutively express glucocorticoid-induced TNFR-related protein (GITR) (45, 46), a member of the TNF receptor crucial to regulate the function of both effector T cells and Tregs during immune responses (47). Although GITR deficiency does not affect homeostasis and suppressive function of Tregs at steady state (48, 49), GITR stimulation on Tregs by agonistic GITR-specific antibody or GITR ligand (GITRL) neutralizes their suppressive capacity to facilitate effector T cell activation and inflammatory responses (45, 46, 50). It has been reported that binding of GITR to GITRL, which is highly expressed in tumors, impairs Treg cell survival and function and consequently enhances antitumor immune responses (45, 51–53). Given that GITRL is frequently expressed in various non-lymphoid tissues, the interaction of GITR/GITRL likely modulates homeostasis and function of tissue Tregs. It is conceivable that diverse TCR and co-stimulatory/co-inhibitory signals constitute a complex network shaping metabolic and functional fitness of tissue Tregs.

### Cytokine Signals

Distinct cytokines have been involved in maintaining homeostasis and function of Tregs in non-lymphoid tissues (54). IL-2 is originally identified as a growth factor that promote expansion and function of effector T cells (55). Tregs constitutively express high levels of the IL-2 receptor CD25 that promotes expansion and function of Tregs. In lymphoid organs, IL-2 selectively maintains homeostasis and function of cTregs, instead of eTregs (3). Several lines of evidence show that a low dose of IL-2 expands and activates Tregs *in vivo* to improve the treatment for a variety of autoimmune diseases (56–58). In line with these observations, the IL-2-signal transducer and activator of transcription 5 (STAT5) axis has been recently determined as a key pathway responsible for Treg expansion *in vivo* (59). Blockade of IL-2 signaling in Tregs leads to accumulation of eTregs (59). IL-7 is a growth factor crucial for homeostatic survival and proliferation of naive CD4^+^ T cells. Accumulating evidence indicates that IL-7 plays an important role in regulating homeostasis and function of Treg cells in tissues. IL-7-transgenic mice and administration of IL-7 enhance expansion of Tregs *in vivo* (17). Using a mouse model of inducible self-antigen expression in skin, a study provided the evidence showing that IL-7 is required to maintain homeostasis and function of skin Tregs (60). In line with the notion, a recent study showed that IL-7 enhances survival and expansion of eTregs and consequently promotes immune tolerance to skin allografts (61). The cytokine IL-33 has been shown to sustain expansion and function of Tregs in a variety of non-lymphoid tissues., a receptor of IL-33. IL-33 promotes expansion and differentiation of VAT-Tregs expressing high levels of the IL-33 receptor ST2. Deletion of ST2 or administration of IL-33 impairs or promotes proliferation of VAT-Tregs (62), respectively. Tregs lacking ST2 compromise their suppressive function in preventing inflammatory responses in the adipose tissue (62). Upon IL-33 stimulation, Basic Leucine Zipper ATF-Like Transcription Factor (BATF) cooperates with interferon regulatory factor 4 (IRF4) to drive transcriptome required to maintain homeostasis and function of VAT-Tregs (62). Furthermore, IL-33-dependent regulation of tissue Tregs has been involved in modulating inflammatory responses under various immune conditions. Lung inflammation induced by acute lung injury is restrained by IL-33-stimulated ST2^+^ Tregs (63). IL-33 enhances expansion and function of skin Tregs in preventing immune responses against skin allografts (64). A recent study revealed that Treg-specific ablation of ST2 exacerbates the progression of EAE (65). Specific blockade of ST2 in Tregs the disease progression (65), indicative of an intrinsic role of IL-33 in Tregs. These studies indicate that diverse cytokines available in the microenvironment are crucial to sustain survival and expansion of tissue Tregs and enhance their function in suppressing inflammatory responses in non-lymphoid tissues.

### mTOR Signaling Pathway

mTOR is an evolutionarily conserved serine/threonine kinase, which exists in two mTOR complexes, mTOR complex 1 (mTORC1) and mTOR complex 2 (mTORC2), distinguished by the essential components regulatory associate protein of mTOR (RAPTOR) and RAPTOR insensitive companion of mTORC2 (RICTOR) (66), respectively. As a central regulator of cell growth and metabolism, mTOR signaling pathway is crucial for homeostatic proliferation, metabolic reprogramming, activation, and suppressive function of Tregs (67). Specific ablation of RAPTOR in Tregs abolishes activation of mTORC1 and consequently compromises their proliferation, activation, metabolic reprogramming as well as suppressive function (68). Raptor-deficient Tregs fail to prevent inflammatory responses in multiple tissues (68). A following study provided the evidence directly indicating that Treg-specific ablation of mTOR disrupts expansion and suppressive function of eTregs, leading to profound inflammation in a variety of non-lymphoid tissues (69). Antigen-experienced Tregs utilize specific amino acids to sustain mTORC1 activation, metabolic reprogramming, and suppressive function (70). Akin to the effects of mTOR deficiency, blockade of availability of these amino acids compromises the generation and function of eTreg cells and impairs their accumulation in non-lymphoid tissues (70). In contrast to the effects of RAPTOR/mTORC1 deficiency on Tregs, loss of RICTOR in Tregs has no substantial impact on their homeostasis and suppressive function at steady state (68). Activation of Treg mTORC2 is tightly regulated by the tumor suppressor PTEN. Treg-specific deletion of PTEN preferentially elevates mTORC2 activation (38), which impairs functional stability of Tregs and consequently promotes the development of autoimmune disorders in multiple tissues (38). Depletion of RICTOR in PTEN-deficient Tregs restores the impaired stability and prevents autoimmunity, implying a therapeutic effect of mTORC2 inhibition. Mice with FOXP3 deficiency develop the scurfy phenotype characterized by profound autoimmunity and inflammation multiple tissues. It is worthy to note that deletion of RICTOR/mTORC2 in FOXP3-deficient Tregs partially restores their augmented aerobic glycolysis and OXPHOS and functional defects (71). Thus, precise regulation of mTORC1 and mTORC2 activation is crucial for Tregs to maintain metabolic fitness and functional stability. Targeting mTORC2 in Treg cells represents an attractive strategy for the treatment of autoimmune diseases.

### LKB1 Signaling Pathway

The serine-threonine kinase liver kinase B1 (LKB1) was originally identified as a tumor suppressor that inhibits mTOR activation and regulates energy metabolism through activating AMP-activated protein kinase (AMPK) and other kinases (72). It was recently identified as a key regulator of Treg cell metabolism and function. Upon TCR stimulation, Tregs show increased phosphorylation of LKB1, deletion of which does not enhance mTOR activation (73). Tregs lacking LKB1 shows impairment of a variety of metabolic programs including mitochondrial OXPHOS and FAO (73, 74), associated with compromised survival function. LKB1-deficient Tregs fail to prevent T_H_2-biased inflammatory responses in a variety of non-lymphoid tissues (73, 74). Genetic deletion of AMPKs has no substantial impact on Tregs (73–76), suggesting that other kinases downstream of LKB1 enforce Treg cell metabolism and function. LKB1 regulates metabolic reprogramming and suppressive function of Tregs through different mechanisms. LKB1 suppresses expression of exhaustion markers including PD-1 through sustaining β-catenin activation (73). Blockade of PD-1 on LKB1-deficient Tregs partially restores their functional defects (73). It has also been shown that Tregs need LKB1 to limit STAT4 activation to enforce their expression of FOXP3 and suppressive function (75). Furthermore, LKB1-deficient Treg cells display increased levels of intracellular cholesterol and the isoprenoid geranylgeranylpyrophosphate (GGPP), indicative of enhanced mevalonate pathway, which impairs Treg cell stability and function (76). It is of great interest to investigate how LKB1 coordinates various metabolic programs and epigenetic modification to orchestrate functional stability and specification of Tregs in maintaining immune tolerance of non-lymphoid tissues.

## Metabolic Signaling in the Regulation of Treg Cell Homeostasis and Function

**Figure 2:**
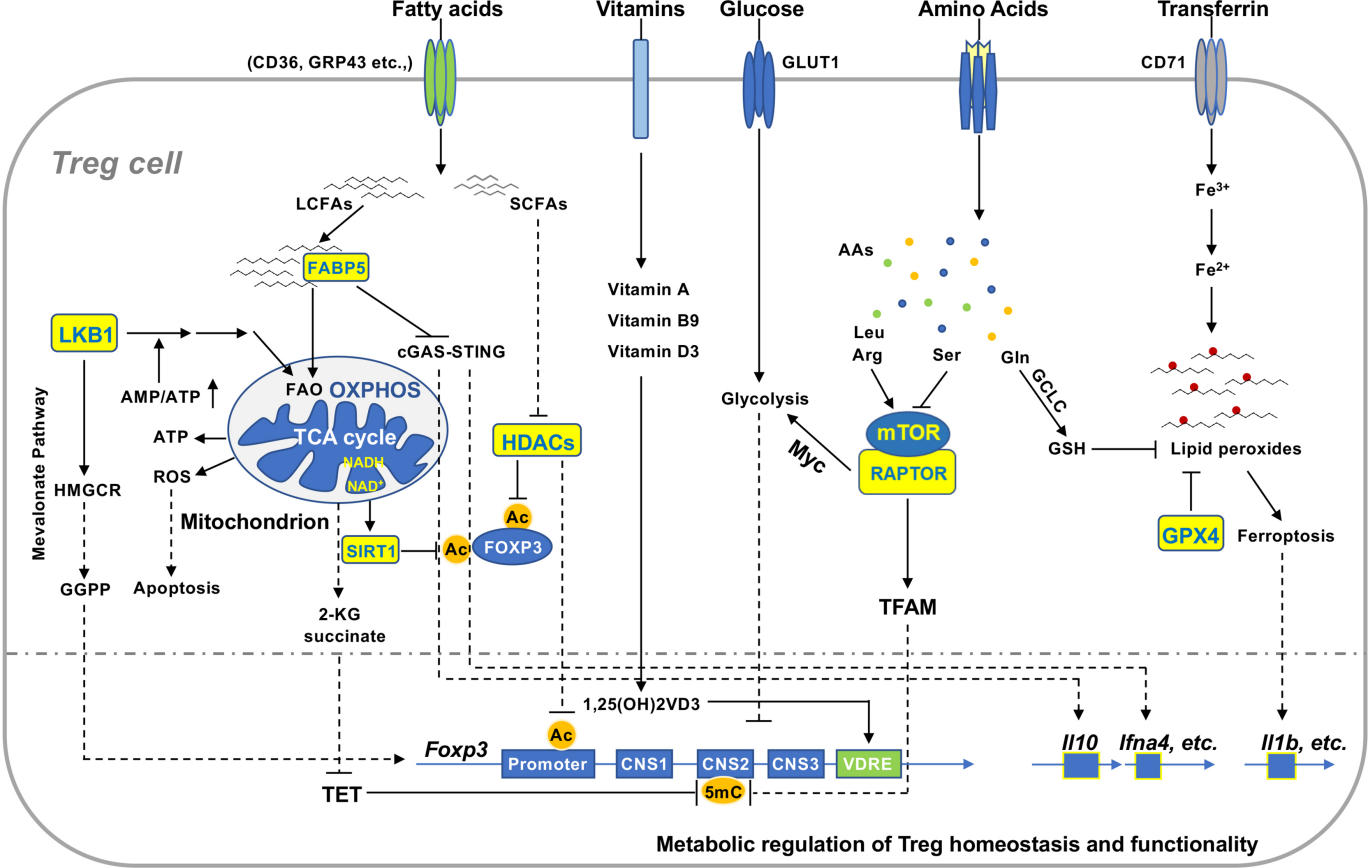
Metabolic signaling in the regulation of Treg homeostasis and function. Extracellular and intracellular metabolites regulate Treg homeostasis and function in various non-lymphoid tissues. Reduced production of ATP activates LKB1 signaling pathway that promotes mitochondrial FAO and OXPHOS and mevalonate pathway producing GGPP, which enhances FOXP3 expression and Treg function. Uncontrolled production of mitochondrial ROS induces Treg apoptosis that facilitates tumor immunoevasion. Increased concentration of cellular NAD^+^ enhances function of the deacetylase SIRT1 that destabilizes FOXP3 protein and compromises Treg function. Tregs take up microenvironmental LCFAs and SCFAs *via* different receptors. FABP5 binds to LCFAs and transfers them to mitochondria in Tregs. Inactivation of FABP5 activates cGAS-STING to promote expression of IL-10 and type I interferons and Treg function. SCFAs suppress activity of HDACs and thereby maintain acetylated FOXP3 and enhance Treg stability. Vitamin D3 metabolite 1,25(OH)_2_VD_3_-receptor complex binds to VDRE region to enhance *Foxp3* gene expression. Distinct amino acids differentially regulate activation of mTOR, which can augment glycolysis to suppress FOXP3 expression *via* Myc or evoke TFAM function to restrain methylation of CNS2. Activated Tregs elevate uptake of transferrin, which can be converted to reductive iron to facilitate the generation of lipid peroxides. GCLC catalyzes the synthesis of GSH crucial for maintaining cellular redox homeostasis. It functions as a substrate for GPX4 to prevent the accumulation of lipid peroxides in Tregs and subsequent ferroptosis. Ferroptotic Tregs enhance production of pro-inflammatory cytokines such as IL-1β. GGPP, geranylgeranylpyrophosphate; ROS, reactive oxygen species; NAD, nicotinamide adenine dinucleotide; LCFAs, long chain fatty acids; SCFAs, short chain fatty acids; HDAC, histone deacetylase; VDRE, vitamin D response element; CNS2, conserved non-coding sequence 2; GCLC, glutamate cysteine ligase catalytic subunit.

Appropriate reprogramming of cellular metabolism is crucial to maintain survival and function of Tregs. The lineage transcription factor FOXP3 of Tregs has been shown to promote OXPHOS and FAO *via* upregulating components of all the mitochondrial electron respiratory complexes while repressing anabolic metabolism (77, 78). A growing body of evidence reveals that metabolites derived from diverse metabolic programs modulate signaling and transcriptional networks in Tregs. Here we summarize key metabolic programs and metabolites underlying Treg cell homeostasis and function (**Figure 2**).

### Mitochondrial OXPHOS and FAO

Mitochondrial respiratory chain complexes play pivotal roles in support of mitochondrial OXPHOS and FAO in Tregs. Suppression of mitochondrial respiratory complex 1 by the small molecule rotenone impairs suppressive function of Tregs (79). Mice with Treg-specific depletion of rieske iron-sulfur protein (RISP), an essential subunit of mitochondrial complex III, spontaneously develop profound inflammation in multiple tissues (80). RISP deficiency disrupts mitochondrial OXPHOS of Tregs and elevates concentrations of intracellular metabolites 2-hydroxyglutarate (2-KG) and succinate, which enhance the methylation of the conserved non-coding sequence 2 (CNS2) in the *Foxp3* locus, by suppressing the ten-eleven translocation (TET) family of DNA demethylases (81). A later study showed that Treg-specific ablation of COX10, a key component of complex VI, impairs mitochondrial OXPHOS and the generation and function of effector Tregs (82). By fueling mitochondrial OXPHOS, FAO provides Tregs with an adequate supply of energy and regulates their differentiation, survival, and function (83, 84). A variety of receptors have been recently shown to mediate uptake of free fatty acids in Tregs, including G protein‐coupled receptors (GPCRs), CD36, fatty acid‐binding protein (FABP), and fatty acid transport protein (FATP) (85, 86). Intracellular LCFAs need to be transported into mitochondria for FAO, which is catalyzed by the rate-limiting enzyme carnitine palmitoyltransferase 1A (CPT1A). Although the CPT1A inhibitor etomoxir has been shown to compromise Treg OXPHOS and function, Treg-specific ablation of CPT1A has no obvious impact on mitochondrial OXPHOS, FAO, and function (82, 87). It is of great interest to further investigate the mechanisms linking mitochondrial FAO and OXPHOS in Tregs.

Several lines of evidence indicate that mitochondrial OXPHOS regulates the adaptation of Tregs to non-lymphoid tissues *via* different molecular mechanisms. Mitochondrial transcription factor A (TFAM) is crucial to regulate mitochondrial DNA replication, transcription, and packaging as well as mitochondrial respiration. Treg-specific depletion of TFAM has no substantial impact on Tregs in lymphoid tissues, while it markedly reduces the differentiation and accumulation of Tregs in a variety of non-lymphoid tissues (81). TFAM-deficient Tregs display impaired OXPHOS and suppressive function (69, 81), associated with hypermethylation of the Treg-specific demethylation region (TSDR) of the *Foxp3* locus (81). It is worth to note that multiple histone/protein deacetylases (HDACs) have been implicated in regulating Treg cell expansion *in vivo* and function (88). In comparison to conventional CD4^+^ T cells, Tregs markedly enhance expression of HDAC9 upon TCR stimulation. Genetic depletion of HDAC9 in Tregs improves mitochondrial OXPHOS through increasing expression of peroxisome proliferator-activated receptor gamma coactivator 1-alpha (PGC1α) and sirtuin-3 (SIRT3) (79). Further studies revealed that Treg-specific depletion of HDAC6 and HDAC11 also sustains their suppressive function (89, 90). Sirtuin1 (SIRT1), a class III histone/protein deacetylase, is activated by increased ratio of NAD^+^ to NADH, which reduces expression of FOXP3 and compromises Treg function (91). Mitochondrial integrity and OXPHOS can be actively regulated by fatty acid binding proteins (FABPs), a family of lipid chaperones that promote lipid uptake and intracellular lipid trafficking. An emerging study revealed that genetic ablation of fatty acid binding protein 5 (FABP5) in Tregs compromises mitochondrial OXPHOS and integrity, leading to release of mitochondrial (mt) DNA into cytoplasm (86). Released mtDNA activates cyclin GMP-AMP synthase (cGAS)-cGAS-stimulator of interferon genes (STING)-dependent type I IFN signaling to enhance IL-10 production and function of FABP5-deficient Tregs (86). These studies imply that mitochondrial metabolism and homeostasis intersects with signaling networks and epigenetic machineries to modulate accumulation and function of Tregs in non-lymphoid tissues.

### Glycolysis

An initial study showed that glycolytic metabolism underlies the differentiation and function of effector CD4^+^ helper T cells but not FOXP3^+^ Tregs (83). Compared to effector CD4^+^ helper T cells, Tregs have less expression of glucose transporter 1 (Glut1). Specific depletion of Glut1 in Tregs blocks their glucose uptake without affecting their suppressive function (92). However, several lines of evidence indicate that glycolysis is required for FOXP3 splicing and regulation of Treg migration and function under different immune conditions. The glycolytic enzyme enolase-1 promotes selective splicing variant of FOXP3 containing exon 2 (FOXP3-E2) in transforming growth factor β (TGFβ)-induced human iTregs upon suboptimal TCR stimulation *in vitro* (93), which regulates Treg suppression of immune responses (93). A recent study highlighted that glucokinase (GCK) regulates Treg cell migration (94). Proinflammatory factors induce GCK-mediated glycolysis in activated Tregs and thereby drive their migration to the inflamed site (94). Interestingly, the PI3K-AKT-mTORC2 axis, but not mTORC1, evokes GCK-dependent glycolysis to support Treg cell migration under inflammatory conditions. In the tumor microenvironment (TME), activated Tregs coordinate active glycolysis and fatty acid biosynthesis to fuel their robust proliferation and enhanced suppressive function (95). In light of these studies, it is conceivable that Tregs integrate diverse immune signals in the tissue microenvironment to modulate glycolytic metabolism in support of their migration and functional fitness.

It is worthy to note that uncontrolled glycolysis is detrimental to Treg cell stability and function. Hypoxia-inducible factor-1 alpha (HIF-1α)-dependnent glycolysis promotes the differentiation of T_H_17 cells and concomitantly suppresses Treg cell generation (96). Several lines of evidence indicate that Treg glycolysis is tightly regulated by various molecular mechanisms. The tumor suppressor PTEN functions as a key metabolic checkpoint in restraining glycolysis and mTORC2 activation in Tregs (38, 97). Loss of PTEN impairs Treg cell stability and function in preventing autoimmune responses in multiple tissues (38, 97). Autophagy has been determined as a key regulator of Treg cell activation and function (98). Treg-specific deletion of autophagy related (ATG) 5 and ATG7, crucial components of autophagy, enhances glycolysis and mTOR activation and consequently promotes profound inflammation in a variety of non-lymphoid tissues (98). Moreover, an emerging study showed that a coactivator of neuronal precursor cell-expressed developmentally downregulated 4 (Nedd4)-family E3 ubiquitin ligase interacting protein 1 (Ndfip1) sustains functional stability of Tregs to prevent inflammatory responses in various tissues (99). Similar to PTEN-deficient Tregs, Tregs lacking Ndfip1 also display enhanced glycolysis and impaired functional stability, while elevating mTORC1 activation (99). A recent study provided evidence indicating that Toll like receptor (TLR) signaling pathways modulate glycolytic activity and functional integrity of Tregs (100). TLR1 and TLR2 agonists enhance Glut1-mediated glycolysis and Treg expansion, while reducing FOXP3 expression and Treg function. These studies imply that precise regulation of glycolysis is crucial for Tregs in maintaining their homeostasis and function in non-lymphoid tissues.

### Lipid Biosynthesis

Accumulating evidence indicates that proper lipid biosynthesis is crucial to ensure Treg cell expansion and functional fitness. Biosynthesis of mevalonate by 25-hydroxycholesterol or 3-hydroy-3-methygltary-CoA reductase (HMGCR) represents a key mechanism coupling Treg cell expansion and suppressive function (68). Blockade of mevalonate biosynthesis in Tregs represses their proliferation and expression of the Treg signatures ICOS and cytotoxic T-lymphocyte associated protein 4 (CTLA4), leading to the failure in preventing inflammatory responses in a variety of non-lymphoid tissues (68). A recent study revealed that mevalonate pathway is also activated by LKB1 signaling pathway, which promotes intracellular cholesterol homeostasis and thereby potentiates Treg cell stability (76). It is interesting to note that LKB1 coordinates multiple metabolic programs including lipid biosynthesis and FAO in Tregs (73, 74) and enforces the suppression of allergic inflammation (73–75), suggestive of beneficial effects of lipid biosynthesis on Treg cell function. In line with the notion, intratumoral Tregs have been shown to elevate lipid biosynthesis in facilitating tumor immunoevasion (42). Treg-specific deletion of sterol-regulatory-element-binding proteins (SREBPs) abolishes fatty acid synthase (FASN)-dependent lipid biosynthesis, which selectively potentiates antitumor immunity without inducing systemic autoimmune disorders (42). It is worthy to note that precise regulation of lipid synthesis is also required to sustain Treg cell functional specification. A recent study provided the evidence indicating that uncontrolled biosynthesis of triglycerides disrupts the function of Tregs in repressing allergic inflammation (101). The transcription factor BATF in Tregs inhibits expression of genes involved in triglyceride biosynthesis. BATF-deficient Tregs show increased levels of intracellular triglycerides. Blockade or elevation of triglyceride metabolism rescues or exacerbates the defective function of BATF-deficient Tregs (101). These studies suggest that proper coordination of biosynthesis of specific lipid species and distinct suppressive programs is important for Tregs in dictating outcomes of different immune responses. Targeting lipid biosynthesis of Tregs represents an attractive strategy for improving the immunotherapy of autoimmune disease and cancer.

### Amino Acids

Amino acids have been identified as immune modulators in shaping the differentiation of Tregs. Tryptophan is a well-documented amino acid involved in the regulation of Treg cell generation and function. The enzyme indoleamine-pyrrole 2,3-dioxygenase 1 (IDO1) expressed by DCs and tumor cells converts tryptophan to kynurenin, which promotes the generation and function of Tregs through the aryl hydrocarbon receptor and other mechanisms (102–106). Emerging evidence indicates that selected amino acids preferentially support the generation and function of eTregs *via* sustaining mTORC1 activation (70). TCR stimulation enhances intracellular concentrations of various amino acids in Tregs, while not all amino acids affect their function and mTOR activation. Arginine (Arg) or the combination of Arg and leucine (Leu) promotes the generation of eTregs through activating mTORC1 dependently of the complex Ras-related GTP binding A/B (RagA/B) (70), which are Rag GTPases mediating translocation of mTORC1 to lysosomal membrane upon amino acid signaling (107). Mice with Treg-specific ablation of RagA/B spontaneously develop severe inflammation in various tissues (70). These findings are in line with the notion that sustained mTOR activation is indispensable for eTreg generation and accumulation in non-lymphoid tissues (68, 69). In contrast, deprivation of Leu alone or glutamine (Gln) or deficiencies of their receptors does not impair the differentiation and proportions of Tregs (108, 109). Non-essential amino acid serine (Ser) supports clonal expansion and function of effector T cells (110). However, Tregs actively restrain uptake or biosynthesis of Ser through glutamate-cysteine ligase catalytic subunit (GCLC) (111), a key enzyme that catalyzes biosynthesis of glutathione (GSH). Treg-specific ablation of GCLC elevates intracellular concentrations of Ser that reduces FOXP3 expression with a concomitant increase of mTOR activation in Tregs, leading to their failure in preventing inflammatory responses in multiple organs (111). In line with the observation, suppression of the cystine/glutamate antiporter solute carrier (SLC)7A11 in human Tregs reduces levels of cellular GSH, associated with increased production of reactive oxygen species (ROS) and diminished mTOR activation (112). Reduced expression of SLC7A11 impairs proliferative capacity of Tregs from the patients with relapsing-remitting multiple sclerosis (RRMS). These findings indicate that distinct amino acids intersect with mTOR signaling pathway to modulate the proliferation and generation of eTregs. It is conceivable that diverse amino acids existing in various non-lymphoid tissues differentially affects homeostasis and accumulation of tissue Tregs.

### Lipid Peroxidation

Upon TCR engagement, activated Tregs reprogram cellular metabolism with increased reliance on mitochondrial OXPHOS and FAO. Enhanced mitochondrial OXPHOS in Tregs potentially elevates the generation of mitochondrial ROS that can disrupt intracellular redox homeostasis (79, 113) and trigger the generation and accumulation of lipid peroxides (114). Various mechanisms have been determined in regulating Treg ROS production, while little is known about the function and role of lipid peroxidation in Tregs. A recent study showed that the glutathione peroxidase plays a pivotal role in neutralizing lipid peroxides generated in activated Tregs and preventing their ferroptosis, an iron-dependent non-apoptotic cell death (115). Despite that Treg-specific ablation of glutathione peroxidase 4 (GPX4) does not affect homeostatic survival of Tregs, TCR stimulation markedly elevates accumulation of toxic lipid peroxides and induces ferroptosis in GPX4-deficient Tregs (115). Ferroptotic GPX4-deficient Tregs release proinflammatory cytokines including IL-1β, which promotes T_H_17 cell responses (115). It is worthy to note that Treg-specific ablation of GPX4 compromises survival of intratumoral Tregs without affecting splenic Tregs from the tumor-bearing mice. Consequently, mice with GPX4-deficient Tregs display reduced tumor burden and concomitantly enhanced antitumor immunity, without showing overt systemic autoimmunity (115). Thus, GPX4 serves as a metabolic checkpoint in preventing accumulation of toxic lipid peroxides and consequent ferroptosis in activated Tregs. The role of GPX4 in regulating homeostasis and function of tissue Tregs needs to be further investigated.

Crosstalk of various metabolic programs shapes the fate decision and function of effector T cells and Tregs in dictating the outcome of different immune responses. Pyruvate dehydrogenase kinase 1 (PDHK1) inhibits the function of pyruvate dehydrogenase (PDH) that converts pyruvate to acyl-CoA to fuel mitochondrial OXPHOS. Th17 cells highly express PDHK1 that selectively promotes aerobic glycolysis and represses OXPHOS, leading to reduced generation of Tregs. Repression of PDHK1 enhances Tregs and alleviates the pathogenesis of autoimmune diseases (84). On the basis of high glycolytic capacity, Th17 cells utilize glucose-derived acetyl-CoA to fuel *de novo* fatty acid synthesis *via* acetyl-CoA carboxylase 1 (ACC1). Suppression of ACC1 blocks this glycolytic-lipogenic metabolic pathway, leading to reduced Th17 differentiation and enhanced Treg generation (116). Elevated glycolysis in Tregs frequently compromises their mitochondrial OXPHOS and functional stability leading to inflammatory and autoimmune disorders (38, 97, 100). It is important to note that crosstalk of glycolysis and lipid biosynthesis exhibits different effects on the function of Tregs in the tumor microenvironment. Emerging evidence showed that intratumoral Tregs enhance glucose uptake to fuel fatty acid synthesis and OXPHOS in support of energetic demands for their expansion and function (95). It is conceivable that dynamic crosstalk of diverse metabolic programs in Tregs confers their homeostasis and functional fitness in various non-lymphoid tissues and immune conditions.

## Diversification of Treg Cell Metabolism and Functional Adaptation to Non-Lymphoid Tissues

Maintaining homeostasis and function of Tregs is indispensable to prevent immune dysregulation over the lifetime. Crosstalk between microenvironmental cues and cellular metabolic networks shapes homeostasis and functional fitness of Tregs. Here we will discuss the mechanisms by which tissue Tregs integrate local immune signals and signaling networks to orchestrate metabolic reprogramming and diverse suppressive programs in adaptation to non-lymphoid tissues.

### Visceral Adipose Tissue

VAT-Tregs play crucial roles in repressing chronic inflammation in the visceral adipose tissue. In comparison to Tregs in lymphoid organs, VAT-Tregs acquire unique features related to lipid metabolism and leukocyte migration (17). Peroxisome proliferator-activated receptor gamma (PPARγ), a crucial regulator of peroxisome-mediated FAO, is highly expressed in VAT-Tregs. PPARγ promotes expression of the genes involved in lipid metabolism, such as CD36, which is a receptor facilitating the import of fatty acids. Treg-specific ablation of PPARγ significantly reduces proportions of VAT-Tregs, without affecting compartment of Tregs in other organs (17). Importantly, loss of PPARγ impairs the function of VAT-Tregs in limiting insulin resistance of high-fat diet (HFD)-administrated mice (17). Several recent studies provided insights into the mechanisms underlying the differentiation and accumulation of Tregs in the visceral adipose tissue. The accumulation of VAT-Tregs need TCR signals derived from MHCII-presented antigens, which are dispensable for their homeostatic proliferation (117). In contrast, administration of the cytokine IL-33 provokes a robust expansion of VAT-Tregs. Conversely, blockade of ST2 decreases the accumulation of VAT-Tregs (117). Further investigation revealed that TCR signaling enhances expression of PPARγ in Tregs (62), while IL-33 drives expansion and accumulation of VAT-Tregs through activating MyD88 signaling pathway to induce BATF- and IRF4-dependent transcriptome (62). In line with the notion, a recent study proposes a two-stage, two-site model to further demonstrate the mechanism underlying acquisition of the distinctive VAT-Treg phenotype (118). By generation of VAT-specific TCR transgenic mice and PPARγ reporter mice, the Mathis group identified a unique subset of splenic Tregs that express low level of PPARγ and acquire a part of the VAT-Treg signature (118). To develop the definitive VAT-Treg phenotype, those cells need to migrate to adipose tissue and receive microenvironmental cues. VAT-specific TCR transgenic mice provide evidence indicating that specific antigens in the adipose tissue are indispensable for accumulation of VAT-Tregs, but not conventional T cells (118). Further, Treg-specific ablation of the IL-33 receptor ST2 diminishes proportions of VAT-Tregs and their FOXP3 expression, but not those in other tissues. IL-33 plays an intrinsic role in driving homeostatic proliferation of VAT-Tregs (118). In light of the rescue effect of IL-33 on VAT-Tregs from obese mice (117, 119), therapeutic administration of IL-33 may enhance the expansion and function of VAT-Tregs in obesity patients. Additionally, other microenvironmental cues, such as IFNα (112), sex hormones (120), and insulin (121), orchestrate homeostasis and function of VAT-Tregs in regulating adipose tissue inflammation and insulin sensitivity.

### Skin

The Skin barrier functions as the first line of defense against external pathogens. Skin Tregs play crucial roles in preserving immune tolerance and sustaining tissue repair in skin (122) and hair regeneration (123). Genetic deletion of Tregs frequently causes profound dermatitis with massive infiltration of T cells in skin (124). Several lines of evidence indicate that homeostasis and function of skin Tregs are regulated through various mechanisms. In comparison to Tregs in lymphoid tissues, skin Tregs secret high levels of the anti-inflammatory cytokine IL-10 (125). Treg-specific deletion of IL-10 does not affect homeostasis of skin Tregs, while it fails to restrain the skin inflammation upon dinitrofluorobenzene (DNFB) challenge (125). It has been appreciated that IL-7 facilitates expansion and function of skin memory Tregs. Antigen‐specific memory Tregs in the skin display higher levels of the IL-7 receptor α chain CD127 than those of splenic Tregs (60) (126),. Blockade of IL-7 singling diminishes accumulation of memory Tregs in skin (60). Moreover, skin Tregs highly express the IL-33 receptor ST2 compared to splenic Tregs (127). ST2 deficiency impairs homeostasis and accumulation of skin Tregs (127), while administration of IL-33 enhances their expansion. It is important to note that a large proportion of skin Tregs expressing GATA-binding protein 3 (GATA-3), the type 2 lineage transcription factor (127–129). Stimulation of TCR signals and IL-33 induces expression of GATA-3 in skin Tregs, which promotes expression of TH2-associated genes to maintain immune homeostasis (127) (130),. Mice with Treg-specific deletion of GATA-3 spontaneously develop a lymphoproliferative disease characterized by TH2-biased inflammation in skin and gut (128, 131, 132). Thus, a diverse range of cytokines sustains homeostasis and functional specification of Tregs in the skin.

Aside from extrinsic effects of local cytokines on skin Tregs, various metabolic programs play intrinsic roles in regulating the adaptation of Tregs to the skin. Human skin Tregs selectively express arginase 2 (ARG2), a mitochondrial enzyme that catalyzes hydrolysis of arginine to ornithine and urea (133). Reduction of ARG2 is associated with impaired function of skin Tregs from psoriatic skin. Overexpression of ARG2 in Tregs enhances their suppressive capacity and accumulation in skin (133). Interestingly, enhanced expression of ARG2 suppresses mTOR signaling in Tregs, which may in turn promote mitochondrial OXPHOS and FAO in skin Tregs (133). The physiological relevance of ARG2 in regulating homeostasis and function of skin Tregs remains to be further investigated. Additionally, 1,25(OH)_2_D_3_ (VitD3), a biologically active metabolite of vitamin D, favors expansion and function of skin Tregs though inducing a specialized subset of dermal dendritic cells (134). Tregs derived from VitD3-primed DCs acquire the capabilities of trafficking to skin and suppressing skin inflammation (134, 135). Further, TX527, a vitamin D analog, enhances suppressive function of human Tregs and polarizes conversion of naive T cells into Tregs, independently of DCs. TX527 promotes migration of human Tregs to skin and their suppression of skin inflammation (135). Collectively, these studies indicate that skin Tregs integrate a wide range of cytokine and TCR signals to orchestrate GATA3-dependent and independent programs in control of skin immune homeostasis. It is of great interest to investigate metabolic mechanisms underlying skin Treg homeostasis and function at steady state and under immune challenges.

### Intestine

Diverse signals influence homeostasis and function of intestinal Tregs to balance pro-inflammatory and anti-inflammatory responses in the gastrointestinal tract. Intestinal Tregs comprise tTregs and peripherally-induced Treg (pTreg) cells, discriminated by their differential expression of Helios and neuropilin 1 (NRP1) (136–138). Cooperation of tTreg and pTregs is crucial to prevent inflammatory responses against harmless dietary antigens and commensal microorganisms (139). Several lines of evidence indicate that microbial and dietary antigens dictate the differentiation and maintenance of intestinal antigen-specific pTregs. Colonic Tregs display heterogeneous repertoires of TCRs, which differ from those used by tTregs and effector CD4^+^ T cells (140). Transgenic expression of TCRs cloned from colonic Tregs on immature thymocytes, followed by adoptive transfer, promotes the differentiation and accumulation of pTregs in the colon, but not in the thymus (140). Importantly, the numbers of colonic Tregs are significantly reduced in germ-free mice and antibiotic-treated mice (136), without diminishing Tregs in the small intestines (136, 141). Later studies revealed that dietary antigens drive the differentiation and accumulation of intestinal Tregs (142). Naive CD4^+^ T cells from transgenic mice of intestinal TCRs preferentially differentiate into pTregs in the small intestine, upon oral exposure to the cognate antigens (143, 144). Thus, persistent TCR signals derived from dietary components and commensal microbiota play indispensable roles in maintaining homeostasis and function of antigen specific Tregs in the gastrointestinal tract.

Local cytokines produced by intestinal Tregs and epithelial cells (IECs) influence Treg cell homeostasis and function. Intestinal Tregs constitutively produce a variety of cytokines including IL-35, TGFβ, and IL-10, in response to diverse antigens derived from commensal microbiota and diets in the gastrointestinal tract. Treg-specific ablation of these cytokines impairs their function in restraining the development of inflammatory-bowel disease (125, 145, 146). The alarmin IL-33 has been shown to regulate homeostasis and accumulation of colonic Tregs expressing ST2. IECs constitutively express IL-33 at steady state, which is notably upregulated under inflammatory conditions (147). IL-33 promotes the differentiation and function of colonic Tregs through inducing GATA3 phosphorylation and activation colonic ST2^+^ Tregs. The IL-33-ST2 axis has been also involved in the regulation of tumor-infiltrating Tregs in colorectal cancer (CRC). A recent study showed that IL-33 promotes function and accumulation of intratumoral Tregs to facilitate immunoevasion of colorectal cancer (148). Genetic ablation of ST2 reduces Treg infiltration and consequently potentiate antitumor immunity to restrain the progression of CRC.

In addition to local antigens and cytokines in the gut, metabolites derived from dietary components and commensal microbes provide crucial signals to shape the differentiation and homeostasis of intestinal Tregs (140). Short chain fatty acids (SCFAs), such as butyrate, propionate and acetate, are products of dietary fiber fermented by specific species of gut bacteria (136, 149). These SCFAs provide key metabolic signaling that underlies homeostasis and function of colonic Tregs through different molecular mechanisms. Butyrate promotes the differentiation and function of colonic Tregs (19, 20, 150), ameliorating the pathogenesis of colitis in Rag1-deficient mice adoptively transferred with CD4^+^ CD45RB^hi^ T cells. Mechanistically, it represses activity of HDACs and thereby enhances histone H3 acetylation in the promoter and CNS regions of the FOXP3 locus (19, 150). Similarly, propionate, but not acetate, inhibits activity of HDACs to promote the differentiation and function of Tregs (19, 150). Several G protein-coupled receptors have been shown to mediate butyrate-induced differentiation and accumulation of colonic Tregs, including free fatty acid receptor 2 (FFAR2, also known as G protein-coupled receptor 43 (GPR43)), GPR109A, and the FFAR2-FFAR3 heteromeric receptor (20, 151, 152).

It is worthy to note that intestinal bile acids (BAs) produced by hepatocytes have been recently shown to modulate homeostasis and function of Tregs expressing retinoic acid receptor-related orphan receptor γ (RORγ) in the colon (153). Despite the importance of RORγ-expressing Tregs in preventing intestinal inflammation (154, 155), elevation of glycolysis can convert them to pathogenic Tregs in facilitating the progression of CRC (156). MondoA is a transcription factor that suppresses glycolysis of colonic Tregs through inducing thioredoxin-interacting protein (TXNIP). Deletion of MondoA in Tregs augments their glycolysis and conversion to IL-17-producing Tregs, which promote CRC progression (156). Thus, food antigens, commensal microbiota, intestinal cytokines, and metabolites constitutes complex networks to orchestrate functional diversity and metabolic fitness of intestinal Tregs in maintaining the balance between protective immunity and immune tolerance in the gastrointestinal tract.

## Metabolic Modulation of Tissue Tregs for Immunotherapy

Given the importance of local nutrients to tissue Tregs, manipulating dietary components has been considered as an attractive strategy targeting Treg cell metabolism and function for immunotherapy. Pre-clinical studies have shown that alteration of certain metabolites in diet effectively influences the differentiation and function of tissue Tregs and improves the treatment for autoimmune diseases and cancer. A variety of vitamins acquired from diet and their metabolites have been shown to promote the suppressive function of Tregs. Vitamin A and its metabolite retinoic acid promotes generation and maintenance of Tregs in the small intestine (144, 157). Vitamin D3 is metabolized to 1,25-dihyroxyvitamin D3 (1,25D) that enhances FOXP3 expression (158). A recent study revealed that mice fed with diets containing vitamin D or 1,25D have increased numbers of colonic RORγ^+^ Tregs with augmented function in repressing dextran sodium sulfate-induced colitis (159). It has been appreciated that vitamin B9 (also known as folic acid) derived from both diets and commensal microbes promotes Treg cell function. Tregs express high levels of the vitamin B9 receptor (folic acid receptor 4; FR4) that sustains their suppressive function in preventing graft rejection or facilitating tumor immunoevasion (160). Vitamin B9 is also required for maintaining survival of colonic Tregs through elevating Bcl2 expression (161). Feeding mice with a vitamin B9-deficient diet selectively reduces numbers of colonic Tregs and predisposes mice to trinitrobenzene sulfonic acid (TNBS)-induced colitis (161).

Accumulating evidence highlights that systemic SCFAs and LCFAs play important roles in shaping homeostasis and function of tissue Tregs. Several lines of evidence indicate that systemic SCFAs affect the suppressive function of tissue Tregs in autoimmune diseases and cancer. An emerging clinical study showed that amounts of the SCFA propionic acid are significantly reduced in serum and feces of patients with multiple sclerosis (MS), an autoimmune and neurodegenerative disease (162). The MS patients supplemented with propionic acid display increased proportions of intestinal Tregs, with a concomitant reduction of T_H_1 and T_H_17 cells. Supplementation of propionic acid also rectifies mitochondrial function of Tregs from MS patients (162). In contrast, augment of serum SCFAs reduces efficacy of the immune checkpoint inhibitor in cancer patients. High levels of serum butyrate and propionate render patents with metastatic melanoma resistant to CTLA-4 blockade (163). Mice supplemented with butyrate-containing water display elevated levels of serum butyrate and increased accumulation of Tregs in the blood and the tumor draining lymph nodes (163). Importantly, butyrate-supplemented mice exhibit reduced antitumor immunity and increased resistance to CTLA-4 blockade (163). Moreover, oleic acid is a monounsaturated 18-carbon LCFA considered as one of the most abundant free fatty acids in various tissues. Emerging evidence indicates that MS patients display reduced concentrations of oleic acid in the blood (164). Oleic acid rectifies impaired mitochondrial OXPHOS metabolism and functional defects of tissue Tregs from MS patients (164). The effects of oleic acid supplementation on the development of MS patients remain to be further investigated. Thus, manipulating systemic metabolites may differentially modulate metabolism and function of tissue Tregs to influence the therapeutic outcome of various immune-related disease.

## Conclusions

It is well accepted that Tregs in non-lymphoid tissues exhibit heterogeneity in their transcriptomes, TCR repertoires, requirements of growth and survival factors, and functional mechanisms. Precise coordination of extracellular milieu and intracellular reprogramming of signaling and metabolic networks is required for tissue Tregs to sustain their homeostasis and suppressive function. In light of advanced understanding of the intersection between cellular metabolism and signaling networks in Treg cell function, manipulating systemic metabolites represents a promising strategy to modulate function of tissue Tregs for the treatment of various autoimmune diseases and tumors. Existing studies have shown that specific antigens and growth factors in the tissue microenvironment function as key regulators of tissue Tregs. However, little is known about the mechanisms of how these factors orchestrate metabolic rewiring of Tregs in adaptation to different non-lymphoid tissues and how distinct tissue metabolites influence homeostasis and function of local Tregs. Given that those factors constitute complex networks in Tregs and among other types of cells in tissues, scRNA-seq of the whole-tissue may provide insight into the identification of novel signaling and metabolic mechanisms involved in the regulation of Treg cell homeostasis and function in tissues. It is important to note that signals derived from other types of cells can shape the suppressive function of tissue Tregs. Cellular indexing of transcriptomes and epitopes by sequencing (CITE-seq) in combination with scRNA-seq holds promise to identify key types of cells and factors that regulate tissue Tregs. Additionally, exploring metabolic profiles in tissue Tregs and distinct tissues may identify novel metabolites that can be manipulated to modulate homeostasis and function of tissue Tregs under different immune conditions.

## Author Contributions

The author confirms being the sole contributor of this work and has approved it for publication.

## Funding

The author acknowledges NIH for the financial support. This work was supported by the National Institute of Allergy and Infectious Diseases (NIAID) Grant No. R01AI53255.

## Conflict of Interest

The authors declares that the research was conducted in the absence of any commercial or financial relationships that could be construed as a potential conflict of interest.

## Publisher’s Note

All claims expressed in this article are solely those of the authors and do not necessarily represent those of their affiliated organizations, or those of the publisher, the editors and the reviewers. Any product that may be evaluated in this article, or claim that may be made by its manufacturer, is not guaranteed or endorsed by the publisher.
